# Cancer-associated fibroblast-secreted glucosamine alters the androgen biosynthesis program in prostate cancer via *HSD3B1* upregulation

**DOI:** 10.1172/JCI161913

**Published:** 2023-04-03

**Authors:** Di Cui, Jianneng Li, Ziqi Zhu, Michael Berk, Aimalie Hardaway, Jeffrey McManus, Yoon-Mi Chung, Mohammad Alyamani, Shelley Valle, Ritika Tiwari, Bangmin Han, Maryam Goudarzi, Belinda Willard, Nima Sharifi

**Affiliations:** 1 Genitourinary Malignancies Research Center, Lerner Research Institute, Cleveland Clinic, Cleveland, Ohio, USA.; 2 Department of Urology, Shanghai General Hospital, Shanghai Jiao Tong University School of Medicine, Shanghai, China.; 3 Metabolomics Shared Laboratory Resource, Lerner Research Institute,; 4 Department of Urology, Glickman Urological and Kidney Institute, and; 5 Department of Hematology and Oncology, Taussig Cancer Institute, Cleveland Clinic, Cleveland, Ohio, USA.

**Keywords:** Oncology, Prostate cancer, Sex hormones

## Abstract

After androgen deprivation, prostate cancer frequently becomes castration resistant (CRPC), with intratumoral androgen production from extragonadal precursors that activate the androgen receptor pathway. 3β-Hydroxysteroid dehydrogenase-1 (3βHSD1) is the rate-limiting enzyme for extragonadal androgen synthesis, which together lead to CRPC. Here, we show that cancer-associated fibroblasts (CAFs) increased epithelial 3βHSD1 expression, induced androgen synthesis, activated the androgen receptor, and induced CRPC. Unbiased metabolomics revealed that CAF-secreted glucosamine specifically induced 3βHSD1. CAFs induced higher GlcNAcylation in cancer cells and elevated expression of the transcription factor Elk1, which induced higher 3βHSD1 expression and activity. Elk1 genetic ablation in cancer epithelial cells suppressed CAF-induced androgen biosynthesis in vivo. In patient samples, multiplex fluorescent imaging showed that tumor cells expressed more 3βHSD1 and Elk1 in CAF-enriched areas compared with CAF-deficient areas. Our findings suggest that CAF-secreted glucosamine increases GlcNAcylation in prostate cancer cells, promoting Elk1-induced *HSD3B1* transcription, which upregulates de novo intratumoral androgen synthesis to overcome castration.

## Graphical abstract

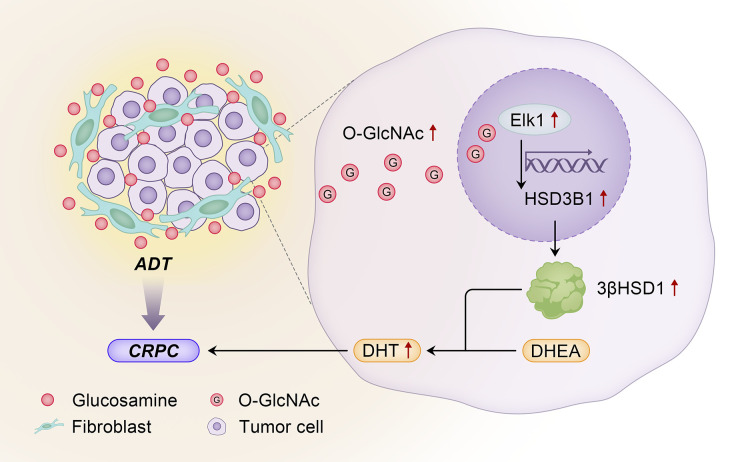

## Introduction

Androgen deprivation therapy (ADT) with medical or surgical castration is the first-line treatment for advanced prostate cancer; it is now often combined with additional therapies (1). However, castration-resistant prostate cancer (CRPC) frequently emerges after the initial clinical response to ADT (2). Androgen receptor (AR) pathway activation is the major driver of CRPC. It is caused in part by intratumoral production of potent androgens (i.e., testosterone and dihydrotestosterone) from extragonadal precursor steroids (3–5). Dehydroepiandrosterone (DHEA), in its free and sulfated forms, is the most abundant circulating precursor for testosterone and/or dihydrotestosterone synthesis (6). Encoded by the *HSD3B1* gene, the enzyme 3β-hydroxysteroid dehydrogenase-1 (3βHSD1) catalyzes the first and rate-limiting step of DHEA metabolism to androstenedione (AD) and downstream androgen synthesis (7, 8). Men who inherit a common germline-encoded missense variation that increases steady-state 3βHSD1 protein more rapidly progress to CRPC and have shorter overall survival compared with men without the variation, as shown across at least 10 clinical cohorts (9–11). Therefore, understanding 3βHSD1 regulation and how it drives resistance and lethality in prostate cancer is vital to strategies that reduce intratumoral androgen production and lead to a sustained treatment response.

Cancer-associated fibroblasts (CAFs) are the most abundant cell type in the tumor microenvironment (12). CAFs play a significant role in CRPC progression and metastasis in large part through paracrine and juxtacrine effects on tumor cells via cytokines such as IL-6, human growth factor, and fibroblast growth factor (13–16). O-GlcNAcylation is a posttranslational modification that attaches *O*-linked *N*-acetylglucosamine to proteins to coordinate nutrient and stress responses (17). Here, we report a potentially unanticipated mechanism in which CAFs induce intratumoral androgen production by 3βHSD1 in prostate cancer cells. We found that glucosamine, a metabolite produced by CAFs, induced high GlcNAcylation, which in turn induced Elk1-mediated transcription of 3βHSD1 in prostate cancer cells. This discovery indicates that CAFs contribute to CRPC progression, not only through production of cytokines and growth factors, but also through secretion of metabolites. Furthermore, targeting Elk1 activity in prostate cancer may decrease intratumoral androgen synthesis to impede CRPC progression.

## Results

### CAF–conditioned medium promotes DHEA-induced AR activation by increasing 3βHSD1 in cancer cells.

**Figure 1:**
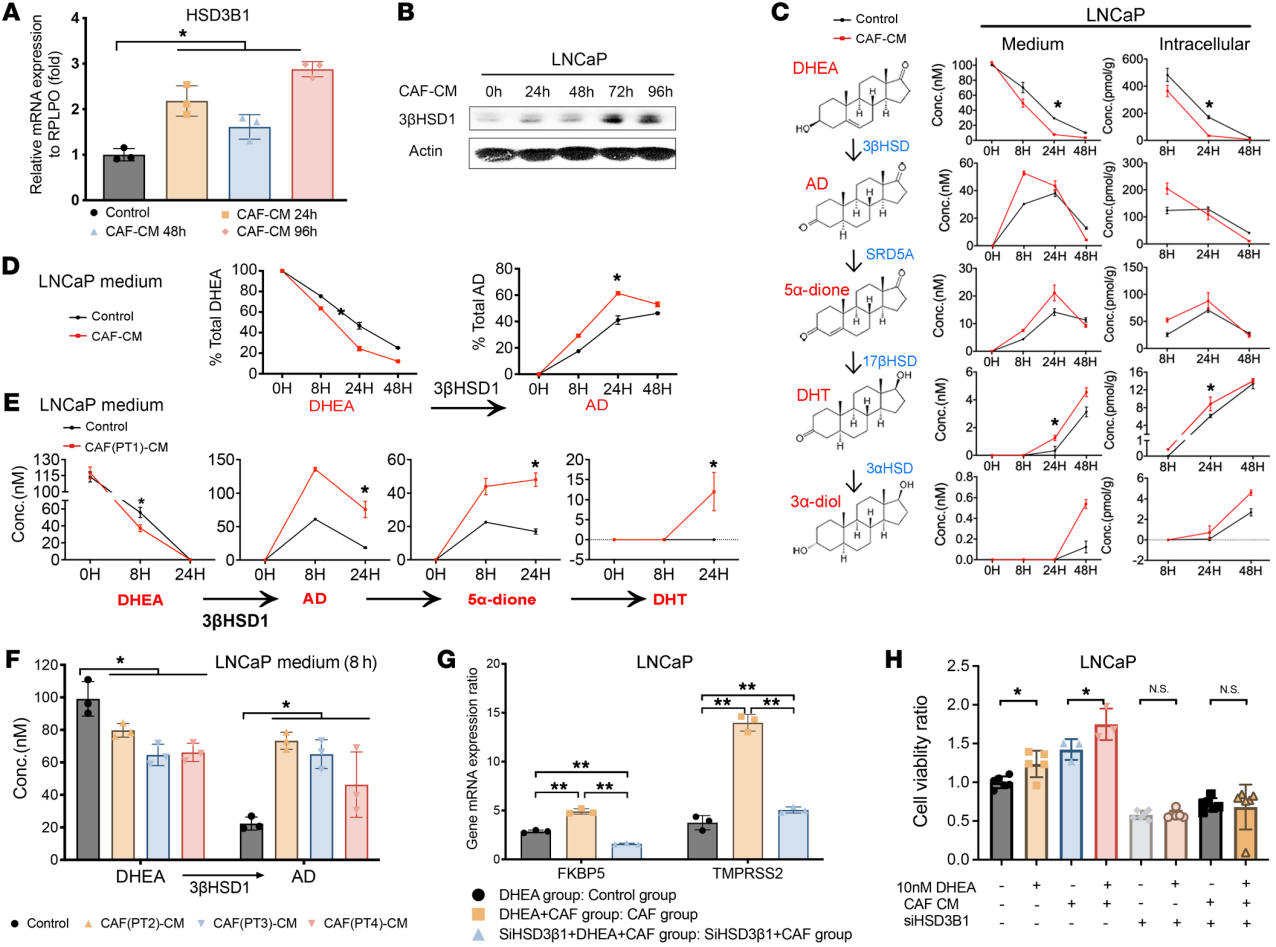
CAFs increase the conversion from DHEA to active androgens in LNCaP cells by increasing 3βHSD1 expression and enzyme activity. (**A**) mRNA (by qPCR) and (**B**) protein expression of *HSD3B1* and 3βHSD1 in LNCaP cells treated with CAF–conditioned medium (CAF-CM) for the indicated times. Gene and protein expression was normalized to RPLP0 and β-actin, respectively. (**C**) LNCaP cells were treated with CAF-CM for 48 hours followed by 100 nM DHEA for the indicated times. Downstream androgens in intracellular and media samples were quantitated by mass spectrometry. (**D**) LNCaP cells were treated with CAF-CM for 48 hours, followed by [^3^H]-DHEA (10^6^ counts per minute) for the indicated times, followed by extraction of steroids from medium and quantitation by HPLC. (**E**) Mass spectrometry analysis of steroids in medium of LNCaP cells treated with DHEA along with CAF-CM derived from primary CAFs isolated from fresh prostate tumor tissue (patient no. 1). (**F**) Mass spectrometry analysis of primary CAF-CM from 3 patients with prostate cancer (patients 2, 3, and 4). (**G**) AR target gene (*FKBP5* and *TMPRSS2*) mRNA expression in LNCaP cells (control or *HSD3B1* siRNA; treated with 10nM DHEA and CAF-CM for 48 hours). Expression was normalized to untreated cells (data not shown), and *RPLP0* was used as a loading control. (**H**) Cell viability of LNCaP control or *HSD3B1* siRNA cells treated with DHEA along with control media or CAF-CM. Viability was normalized to the untreated control. Unless otherwise noted, data are shown as mean ± SEM. Significance was calculated using 2-tailed *t* tests or 1-way ANOVA as appropriate. *P < 0.05, **P < 0.01. Conc., Concentration.

To investigate how CAFs regulate androgen metabolism in prostate cancer, we determined the effect of CAF–conditioned medium (CAF-CM) treatment on 3βHSD1 expression in the LNCaP and C4-2 human cell line models of prostate cancer. CAF-CM treatment increased *HSD3B1* mRNA and 3βHSD1 protein expression in a time-dependent manner (Figure 1, A and B, and Supplemental Figure 1, A and B; supplemental material available online with this article; https://doi.org/10.1172/JCI161913DS1). To determine the functional metabolic effects of 3βHSD1 induction, metabolic flux from DHEA to downstream androgens was assessed by mass spectrometry in cells and media. CAF-CM treatment increased conversion from DHEA to downstream androgens in both prostate cancer cell line models (Figure 1C and Supplemental Figure 1C). Similarly, incubation with CAF-CM increased [^3^H]-DHEA metabolism to [^3^H]-AD (Figure 1D and Supplemental Figure 1D). Additionally, media from primary CAF cultures that were derived from tissues from patients with prostate cancer promoted DHEA metabolic conversion in LNCaP cells (Figure 1, E and F). More importantly, in the CAF-CM environment, DHEA strongly activated AR-responsive genes in both prostate cancer cell lines; activation was attenuated by *HSD3B1* siRNA (Figure 1G and Supplemental Figure 1E). Similarly, CAF-CM treatment increased cancer cell viability after DHEA treatment, and *HSD3B1* siRNA impeded this effect (Figure 1H and Supplemental Figure 1F). Together, these data show that component(s) of CAF-CM increase prostate cancer conversion from DHEA to downstream potent androgens via 3βHSD1.

### Glucosamine in CAF-CM contributes to increased HSD3B1 expression and more rapid DHEA metabolism.

To identify the nature of the CAF-secreted factors that alter steroid metabolism in cancer cells, we subjected conditioned medium to 3 freeze-thaw cycles (–80°C/50°C), boiling (100°C, 15 min), or pronase digestion and observed that it retained the ability to increase 3βHSD1 expression and enzyme activity (Supplemental Figure 2, A–C), indicating that the responsible factor(s) probably lacked tertiary structure. Moreover, the activity was retained in medium passed through a 3 kDa cutoff filter (Supplemental Figure 2D). The filter size and resistance to extreme temperatures excluded larger molecules, such as proteins, suggesting that a small molecule/metabolite(s) mediates the effect on androgen biosynthesis.

**Figure 2:**
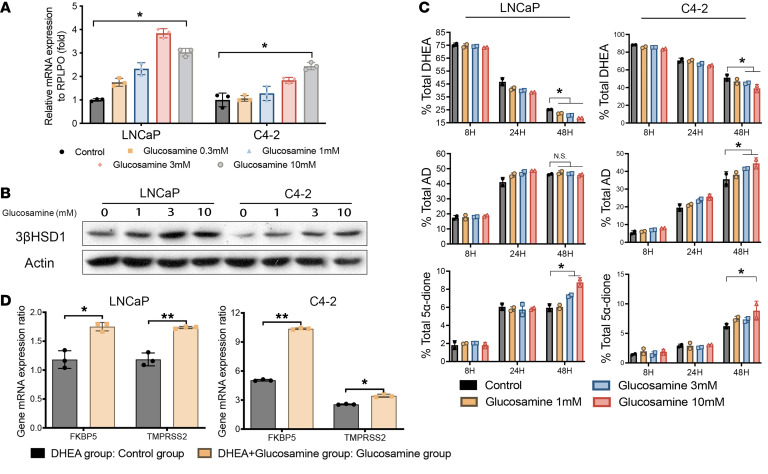
Glucosamine in CAF-CM induces *HSD3B1* expression and the androgen metabolism phenotype. (**A**) mRNA (qPCR) and (**B**) protein expression of 3βHSD1 in LNCaP and C4-2 cells treated with increasing concentrations of glucosamine for 48 hours. Significance was calculated using 2-tailed *t* tests (control versus 10 mM glucosamine). (**C**) HPLC analysis of steroids in media of C4-2 and LNCaP cells treated with the indicated concentrations of glucosamine for 48 hours and [^3^H]-DHEA (1,000,000 counts per minute) for the indicated times. Significance was calculated at 48 hours using 1-way ANOVA. (**D**) Gene expression of AR target genes in C42 and LNCaP cells treated with 10 nM DHEA in the presence or absence of 10 mM glucosamine. Data were normalized to *RPLP0,* and significance was calculated using 2-tailed *t* tests. *P < 0.05.

We next performed a series of metabolomic profiling analyses to identify possible metabolites in CAF-CM that may account for the changes in androgen metabolism (Table 1). We validated these findings by treating LNCaP and C4-2 cells with the top candidate metabolites in Table 1. Of the top putative metabolites that are commercially available, including lactosamine, N-glutarylglycine, azelaic acid, glucosamine, galactosamine, fructosamine, N-acetyl-L-alanine, and hydroxyproline (negative data not shown), only glucosamine increased 3βHSD1 expression and enzyme activity in cancer cells (Figure 2, A–C). Moreover, glucosamine was preferentially produced by CAF (Supplemental Figure 3) and activated the AR pathway in cells treated with 10 nM DHEA as compared with those treated only with DHEA (Figure 2D). Conditioned media from normal prostate fibroblasts did not appear to have the same effect as CAFs on DHEA metabolism (Supplemental Figure 4).

### CAF-CM/glucosamine induces 3βHSD1 expression and enzyme activity by increasing O-GlcNAcylation.

**Figure 3:**
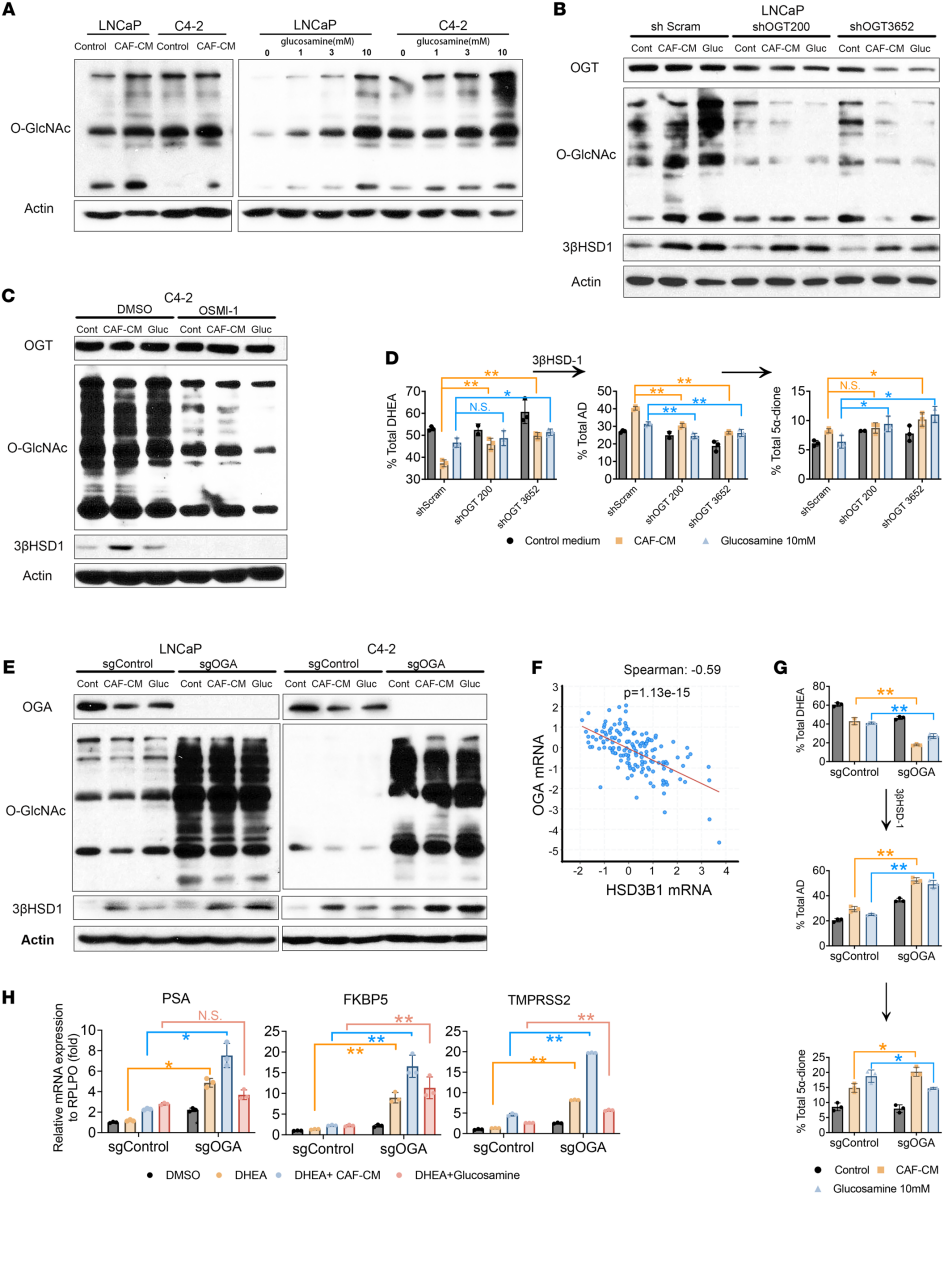
O-GlcNAcylation after CAF-CM treatment is attributable to glucosamine and induces 3βHSD1 expression and enzyme activity. (**A**) Western blot analysis of O-GlcNAcylated proteins in LNCaP and C42 cells treated with CAF–conditioned medium (CAF-CM) (left) or increasing concentrations of glucosamine (right) for 48 hours. (**B**) Western blot analysis of O-GlcNAcylated proteins, OGT, and 3βHSD1 protein expression in LNCaP cells expressing 2 shRNAs targeting OGT (OCG200 and OGT3652) or scrambled shRNA and treated with CAF-CM or 10 mM glucosamine for 48 hours. (**C**) O-GlcNAcylated proteins, OGT, and 3βHSD1 protein expression in C4-2 cells treated with CAF-CM or 10 mM glucosamine in the presence of DMSO (vehicle control) or 75 μM OSMI-1, an OGT inhibitor. (**D**) HPLC analysis of DHEA metabolism in LNCaP cells expressing scrambled shRNA, shOCG200, and shOGT3652 treated with CAF-CM or 10 mM glucosamine for 48 hours, followed by addition of [^3^H]-DHEA (100 nM) for the indicated times. Significance was calculated using 1-way ANOVA. (**E**) Western blot analysis of OGA, O-GlcNAcylated proteins, and 3βHSD1 expression in LNCaP (left) and C4-2 (right) cells transduced with control (sgControl) or sgRNA targeting OGA (sgOGA) after 48-hour treatment with CAF-CM or 10 mM glucosamine. (**F**) Pearson correlation analysis of *HSD3B1* and *OGA* mRNA expression in prostate cancer (MSKCC Prostate Oncogenome Project, GSE21032). (**G**) HPLC analysis of DHEA metabolism in media of LNCaP control or OGA-KO cells treated with CAF-CM or glucosamine for 48 hours, followed by [^3^H]-DHEA (100 nM) for the indicated times; data were normalized to untreated control. (**H**) Gene expression of AR target genes PSA, FKBP5, and TMPRSS2 in control and *OGA*-KO LNCaP cells treated with CAF-CM or 10 mM glucosamine plus 10 nM DHEA for 48 hours. Significance was calculated using 2-tailed *t* tests. *P < 0.05, **P < 0.01.

In cancer cells, glucosamine has been reported as a substrate for protein O-GlcNAcylation by O-GlcNAc transferase (OGT) (17), and O-GlcNAcase (OGA) can remove the O-GlcNAc modification. O-GlcNAcylation has been reported in nearly all cancers examined and can regulate many hallmark characteristics of cancer, including growth, survival, metabolism, angiogenesis, and metastasis (18). To determine the mechanism underlying our observed effect of glucosamine and CAF-CM on AR signaling, we first analyzed changes in O-GlcNAcylation in LNCaP and C4-2 cells. As expected, treatment with either CAF-CM or glucosamine greatly increased the overall level of O-GlcNAcylation (Figure 3A).

After knocking down OGT with stable shRNA expression or using a selective OGT inhibitor (OSMI-1), we found that GlcNAcylation induced by CAF-CM or glucosamine was inhibited, as was 3βHSD1 expression (Figure 3, B and C, and Supplemental Figure 5A). Moreover, OGT shRNA impeded the upregulation of DHEA metabolism by CAF-CM or glucosamine treatment (Figure 3D and Supplemental Figure 5B).

In contrast, KO of OGA increased O-GlcNAcylation, and expression levels of 3βHSD1 were markedly elevated by sgRNA KO of OGA or CAF-CM or glucosamine treatment (Figure 3E). In addition, cBioPortal analysis of a clinical data set showed that OGA mRNA levels inversely correlated with *HSD3B1* mRNA levels in prostate cancer tissues (Figure 3F). Consistent with mRNA expression, 3βHSD1 enzyme activity also increased in OGA-KO cells treated with CAF-CM or glucosamine (Figure 3G and Supplemental Figure 5C). Moreover, in the CAF-CM or glucosamine environment, 10 nM DHEA increased mRNA expression of AR target genes after OGA KO (Figure 3H). Together, these data show that CAF-generated glucosamine increases metabolic flux from DHEA to downstream androgens in prostate cancer by way of O-GlcNAcylation.

### High O-GlcNAcylation elevates the transcription factor Elk1, which induces higher 3βHSD1 expression and enzyme activity.

**Figure 4:**
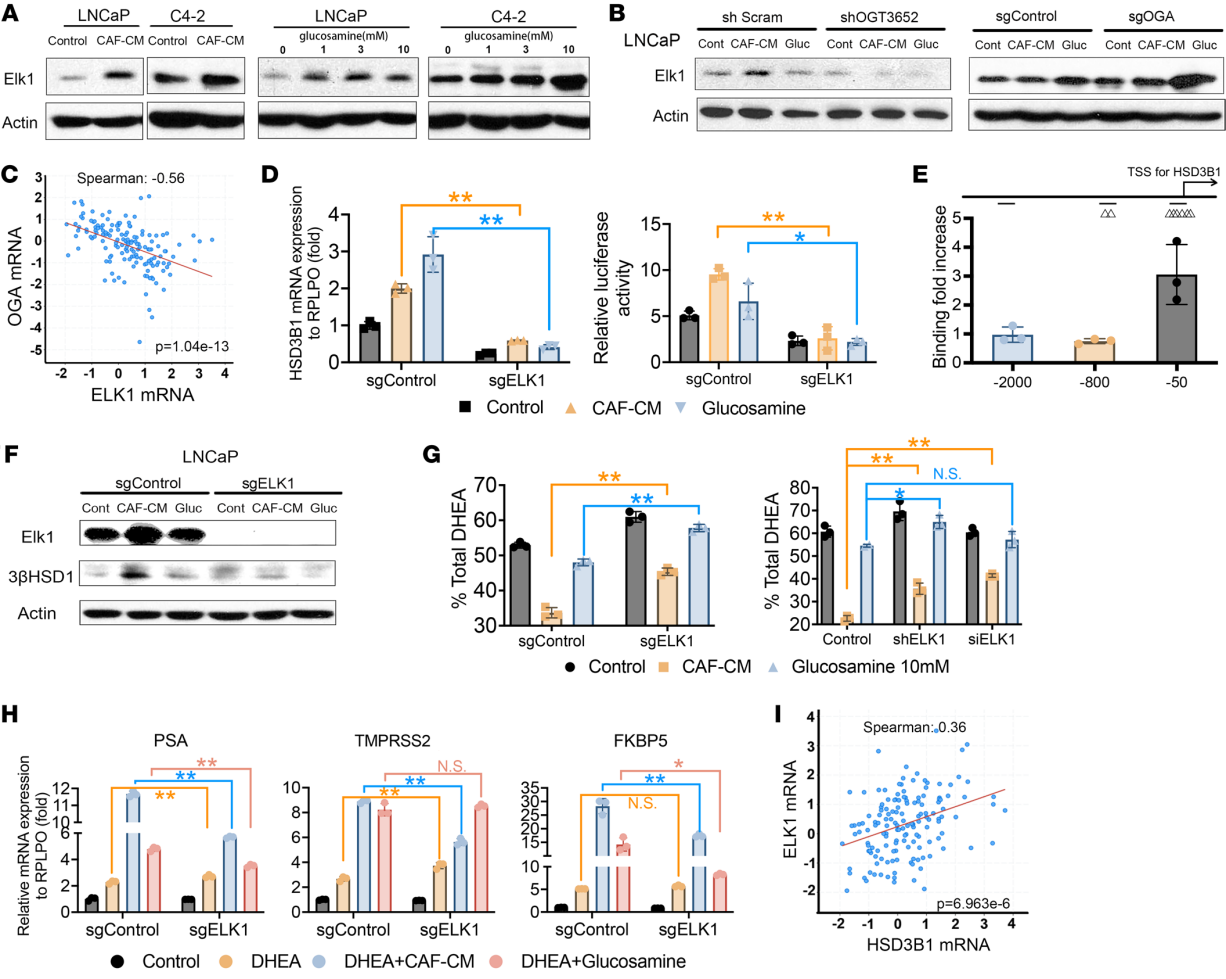
High O-GlcNAcylation increases Elk1 to induce 3βHSD1 expression and enzyme activity. (**A**) Protein expression of ELK1 in LNCaP and C42 cells treated with CAF–conditioned medium (CAF-CM) (left) and increasing concentrations of glucosamine (right) for 48 hours. (**B**) ELK1 protein expression in LNCaP cells expressing control or OGT shRNA (left) and sgRNA (right) treated with CAF-CM or 10 mM glucosamine for 48 hours. (**C**) Pearson correlation analysis of *ELK1* and *OGA* mRNA expression in prostate cancer (GSE21032). (**D**) Left: Gene expression of *HSD3B1* in control (sgControl) or ELK1-KO (sgELK1) LNCaP cells treated with CAF-CM or 10 mM glucosamine for 48 hours. Right: Luciferase assay of LNCaP control and ELK1-KO cells cotransfected with an *HSD3B1* promoter-firefly luciferase and *Renilla* luciferase plasmid constructs, which were treated with CAF-CM or 10 mM glucosamine 48 hours. (**E**) ChIP assay of Elk1. C4-2 cells were treated with 10 mM glucosamine for 24 hours. (**F**) Protein expression of ELK1 and 3βHSD1 in sgControl and *ELK1*-KO LNCaP cells treated with CAF-CM or glucosamine. (**G**) HPLC analysis of steroids in media of LNCaP cells expressing sgControl and *ELK1* KO (left) or siRNA and shRNA knockdown of ELK1 (right). Cells were treated with CAF-CM or glucosamine for 48 hours, followed by [^3^H]-DHEA (100 nM) for 48 hours. (**H**) Gene expression of *PSA*, *TMPRSS2*, and *FKBP5* in sgControl and *ELK1*-KO LNCaP cells treated with 10 nM DHEA along with CAF-CM or glucosamine for 48 hours. (**I**) Pearson correlation analysis of *ELK1* and *HSD3B1* mRNA expression in prostate cancer (GSE21032). Significance was calculated using 2-tailed *t* tests. *P < 0.05, **P < 0.01.

ChIP-Seq in LNCaP cells has shown that O-GlcNAc sites have the typical chromatin structure of a region bound by transcription factors, and the top motif for O-GlcNAc ChIP-Seq is similar to the ELK1 motif (19). ELK1 also was the top transcription factor that potentially regulated O-GlcNAc–marked genes after OGT inhibitor treatment (19). Therefore, we determined whether Elk1 activation is the mechanism underlying the O-GlcNAcylation–induced phenotype. Indeed, both CAF-CM and glucosamine increased Elk1 expression in LNCaP and C4-2 cells (Figure 4A). In the presence of CAF-CM or glucosamine, Elk1 expression levels were consistent with the changes in O-GlcNAcylation (Figure 3B) after genetically blocking OGT or OGA expression (Figure 4B). Moreover, cBioPortal analysis showed that OGA and ELK1 mRNA levels inversely correlated (Figure 4C). However, we did not find that Elk1 was O-GlcNAcylated in cancer cells (Supplemental Figure 6A).

After ELK1 KO in LNCaP cells, CAF-CM– or glucosamine-induced HSD3B1 mRNA expression was reduced, and an *HSD3B1* promoter firefly reporter system also showed decreased luciferase activity (Figure 4D). A ChIP assay indicated that Elk1 binds to the –50 bp region of the *HSD3B1* transcriptional start site (Figure 4E). After glucosamine treatment, increased O-GlcNAc and acetylation on histones may increase chromatin accessibility and facilitate Elk1 binding to the promoter (Supplemental Figure 7). As expected, CAF-CM– or glucosamine-induced increases in 3βHSD1 protein and enzyme activity were both impeded in ELK1-KO cells (Figure 4, F and G). ELK1 KO reduced the increase in AR pathway gene mRNA (*PSA*, *TMPRSS2*, *FKBP5*) expression seen with 10 nM DHEA and CAF-CM or glucosamine treatment (Figure 4H). Results in C4-2 cells were similar (Supplemental Figure 6, B–E). Consistent with Elk regulation of *HSD3B1*, analysis of the clinical expression sets showed that *HSD3B1* and *ELK1* mRNA levels positively correlated (Figure 4I).

**Figure 5:**
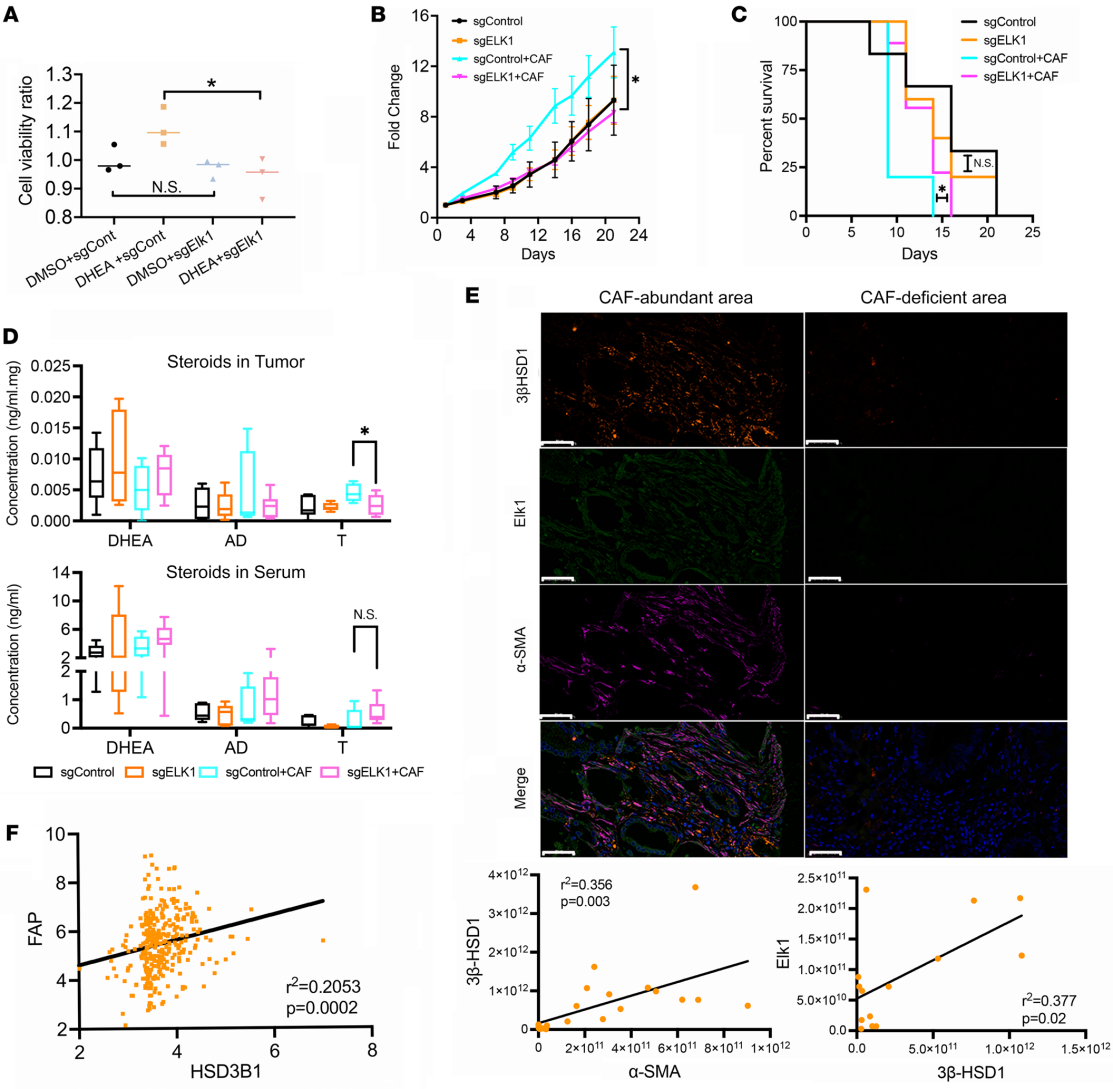
Elk1 induced 3βHSD1 expression and DHEA metabolism in vivo. (**A**) Cell viability of sgControl and *ELK1*-KO LNCaP cells treated with DMSO (control) or 10 nM DHEA for 48 hours. (**B**) Xenograft tumor growth of orchiectomized mice subcutaneously injected with control or *ELK1*-KO C42 cells in the absence or presence of CAFs. A 2-tailed paired *t* test was performed between control and *ELK1*-KO tumors coinjected with CAFs at day 21. (**C**) A log-rank test was used to compare progression-free survival between control and *ELK1*-KO and C4-2 cells grown with CAFs. (**D**) Mass spectrometry analysis of intratumoral and serum DHEA, AD, and testosterone (T) in control or *ELK1*-KO C42 cells. (**E**) Representative multiplexed fluorescence image of a patient with prostate cancer (Gleason 4+4). 3βHSD1, orange; Elk1, green; CAF, α-SMA, purple), and DAPI, blue. Scale bar: 50 μm. Pearson correlation analysis of gene expression in tissues from patients with primary prostate cancer (3βHSD1 and CAF [α-SMA], *n* = 22; 3βHSD1 and Elk1, *n* = 14). (**F**) Pearson correlation analysis of *HSD3B1* and *FAP* mRNA (CAF) in human CRPC metastases (GSE77930). Unless otherwise noted, data are shown as mean ± SEM. Significance was calculated using a 2-tailed *t* test or 1-way ANOVA. *P < 0.05, **P < 0.01.

To determine whether Elk1 regulates cancer cell growth, we conducted in vitro and mouse xenograft studies with control LNCaP and ELK1-KO cells. We found that loss of Elk1 did not change LNCaP cell growth under hormone-free conditions, but it did reduce growth of LNCaP cells treated with DHEA (Figure 5A). Additionally, Elk1 loss had no effect on tumor growth or progression in the absence of CAFs in vivo. In contrast, in the absence of Elk1, C4-2 xenograft tumor volume in mice treated with orchiectomy and DHEA pellet implantation (to mimic human adrenal physiology) (20–22) was significantly lower in the presence of CAFs and resulted in increased progression-free survival (Figure 5, B and C). Moreover, in the presence of both CAFs and Elk1 loss, testosterone was lower in tumor tissue, whereas no change in serum testosterone was detected (Figure 5D).

**Figure 6:**
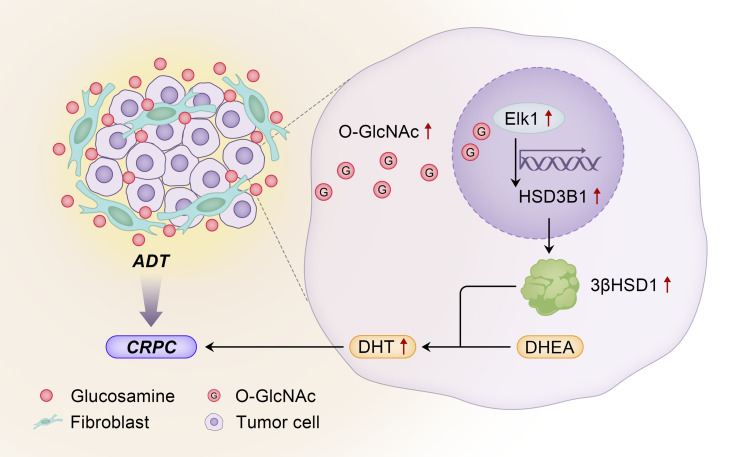
The physiology and mechanisms by which glucosamine originating from fibroblasts induces androgen biosynthesis and resistance in prostate cancer. Cancer-associated fibroblasts synthesize and secrete glucosamine in the tumor microenvironment. In the prostate cancer tumor cell, glucosamine induces an increase in O-GlcNAc, which in turn elicits Elk1-dependent transcription of *HSD3B1*. *HSD3B1* is translated to its corresponding enzyme, 3βHSD1, which is the rate-limiting step for prostate cancer to convert adrenal DHEA to the potent androgen, DHT, to promote progression to castration-resistant prostate cancer (CRPC).

To assess the translational impact of our findings, we assessed changes in 3βHSD1 expression in patient prostate cancer samples. Multiplexed fluorescence staining showed that in primary tumor areas where CAFs were enriched, 3βHSD1 expression and Elk1 expression were increased. In the CAF-deficient areas, expression of both 3βHSD1 and Elk1 was lower. Analysis of the clinical samples showed that 3βHSD1 and Elk1 expression positively correlated (Figure 5E). mCRPC patient data (GEO GSE77930) also showed that *HSD3B1* and *FAP* mRNA levels were positively correlated (Figure 5F). These data suggest that CAF-secreted glucosamine alters androgen metabolism in tumor cells by inducing ELK1 regulation of 3βHSD1 (Figure 6).

## Discussion

After an initial positive response to ADT, metastatic prostate cancer recurs as CRPC, which is responsible for almost all prostate cancer deaths. A major mechanism underlying CRPC is activation of the AR signaling axis. A multitude of AR stimulation mechanisms have been described, including AR gene and enhancer amplification, AR mutation, coactivator overexpression, and intratumoral de novo androgen synthesis, among others (2, 23, 24). The clinical survival benefit conferred by abiraterone, an inhibitor of extragonadal androgen synthesis, clearly demonstrates a major role for intratumoral androgen synthesis in driving CRPC (25–28). Of note, physiologically significant intratumoral androgens in the presence of castration derive largely from the adrenal precursor DHEA, which requires the enzymatic action of 3β-HSD1, encoded by *HSD3B1* (9). Previously, we found that a missense-encoding single nucleotide polymorphism (1245A>C) in *HSD3B1* stabilizes the protein and subsequently increases DHT synthesis from DHEA. This more active form of 3β-HSD1 is inherited in about half of all patients with CRPC and drives more aggressive clinical outcomes and shorter overall survival after treatment with ADT, thus providing genetic evidence for *HSD3B1* in promoting resistance in clinical prostate cancer (7, 29, 30). Here, we demonstrate that CAFs in the tumor microenvironment can increase *HSD3B1* transcription to promote CRPC progression. This observation moves us closer to an understanding of *HSD3B1* transcriptional regulation and how androgen metabolism is fine-tuned at the level of the prostate tumor microenvironment.

Our studies identified glucosamine as a key metabolite produced by CAFs responsible for increased 3β-HSD1 enzymatic activity. Glucosamine is metabolized by the hexosamine biosynthetic pathway, whose end product is uridine diphosphate N-acetyl glucosamine (UDP-GlcNAc). UDP-GlcNAc serves as a basis for posttranslational modifications, including O-GlcNAcylation, which conjugates this sugar to a wide variety of proteins, including metabolic enzymes, transcription factors, and signaling molecules (18). Our results indicate that the glucosamine-mediated increase in 3β-HSD1 requires O-GlcNAcylation. Specifically, higher O-GlcNAcylation in tumor cells treated with glucosamine induced Elk1-mediated transcription of *HSD3B1* and increased tumor cell viability. A role for enhanced hexosamine biosynthetic pathway activity and O-GlcNAcylation in PCa has been demonstrated (19, 31–33), and our discovery mechanistically links O-GlcNAcylation to sustained androgen metabolism and thus CRPC. However, we did not directly assess the glucosamine secretory mechanism in CAFs.

Chronic dysregulation of O-GlcNAcylation plays a role in the progression of other pathophysiological conditions, including diabetes (18). Numerous clinical studies have identified positive associations between metabolic abnormalities, including hyperglycemia and diabetes, and PCa aggressiveness and recurrence following ADT (34–39). The mechanisms underlying these associations are elusive. Our data suggest that systemic glucosamine and/or glucose plays a mechanistic role. Indeed, serum glucosamine has been found to be elevated in patients with type 2 diabetes in metabolomics studies (40, 41). It is also possible that the hyperglycemia associated with diabetes has an effect similar to glucosamine on O-GlcNAcylation–induced 3β-HSD1 activity, as glucose is a primary substrate of the hexosamine biosynthetic pathway (18). Because ADT in men with PCa has the potential to cause metabolic dysfunction (42), it may be that elevated systemic glucose/glucosamine accompanying ADT can act to enhance intratumoral androgen synthesis and ultimately drive treatment resistance.

Our study also identified a potentially novel role for Elk1 in promoting *HSD3B1* transcription with increased O-GlcNac levels. Elk1 is reported to be a strong, independent prognosticator of PCa recurrence, according to TCGA database analysis (43). Although the mechanism remains to be determined, upregulated *HSD3B1* transcription by Elk1 could explain, at least in part, why Elk1 is a robust predictor of recurrence.

In addition to its role in prostate cancer, 3βHSD1 plays an essential role in estrogen-driven breast cancer — particularly in postmenopausal women. Specifically, 3βHSD1 is 1-step upstream of aromatase and is therefore essential for conversion of DHEA to estradiol and estrone. The homozygous adrenal-permissive *HSD3B1* genotype is enriched in ER-positive postmenopausal breast cancer (44). Furthermore, in a cohort of over 600 women with postmenopausal ER-positive breast cancer, those who inherited the homozygous adrenal-permissive *HSD3B1* genotype had a 5-fold elevated risk of metastatic recurrence compared with women who did not inherit the adrenal-permissive allele (45). This genetic evidence for *HSD3B1* in driving breast cancer therefore suggests that the fibroblast-glucosamine-3βHSD1 axis may also play an essential role in breast cancer pathogenesis. However, this remains to be explored.

Overall, our findings suggest that CAFs in the tumor microenvironment upregulate de novo androgen synthesis within tumor cells to overcome castration. CAF production of glucosamine increases GlcNAcylation in cancer cells, promoting Elk1-induced *HSD3B1* transcription.

## Methods

### Cell culture and conditioned medium.

LNCaP, C4-2, prostate cancer CAFs, and normal prostate fibroblasts were purchased from ATCC, cultured in RPMI-1640 medium containing 10% FBS and 1% penicillin-streptomycin (Cleveland Clinic Media Core), and switched to RPMI-1640 with 10% charcoal-stripped FBS prior to experimental treatments.

Patient-derived primary CAFs were established and characterized as previously described (46, 47). In brief, specimens were minced and suspended in RPMI-1640 medium–containing collagenase (1 mg/mL) and DNase (1 mg/mL) at 37°C for 24 hours. The suspension was filtered and separated by a gradient technique. The upper cells were washed and cultured in RPMI-1640 medium–containing 20% FBS at passage 1, and passages 3–8 were used for experiments performed with RPMI-1640 medium and 10% charcoal-stripped FBS.

CAF-CM and conditioned media from normal prostate fibroblasts were produced by seeding 10^6^ CAFs per normal prostate fibroblasts in 10 cm well plates for 24 hours. Medium was replaced after 24 hours, harvested after 48 hours, and then filtered using a 0.2 μM filter and stored at –80°C.

Protein-denatured conditioned medium was obtained by 3 freeze-thaw cycles (–80°C/50°C), boiling (100°C, 15 min), or pronase digestion (Sigma-Aldrich). The protein-denatured medium was then subjected to centrifugal filtration (3 kDa molecular weight cutoff; Merck) for use in conditioned medium experiments.

### Plasmids, siRNA, and chemicals.

Control siRNA, siHSD3B1, and siELK1 SMARTpool were purchased from Dharmacon. Cells were seeded at 60%–80% confluence and transfected using Lipofectamine RNAiMAX for 48 hours according to the manufacturer’s instructions (Life Technology). Cells were then used for experiments.

Control OGA (nos. 1733, 1738, 2246) and ELK1 (nos. 1326, 1327, 1663) gRNA plasmids were purchased from VectorBuilder. Control shRNA, shOGT (TRCN0000286200, TRCN0000293652), and shELK1 (TRCN0000007451) plasmids were purchased from Sigma-Aldrich. These plasmids were cotransfected with lentiviral packaging vectors psPAX2 and pMD2.G into 293T cells, and viral particles were collected for cancer cell infection. LNCaP and C42 cells were positively selected using puromycin (5 μg/mL) for 1 week.

The O-GlcNac inhibitor αR-[[(1,2-dihydro-2-oxo-6-quinolinyl)sulfonyl]amino]-N-(2-furanylmethyl)-2-methoxy-N-(2-thienylmethyl)-benzeneacetamide (OSMI-1) was purchased from Sigma-Aldrich.

### Gene expression and immunoblot.

Total RNA was extracted with a GenElute Mammalian Total RNA miniprep kit (Sigma-Aldrich), and 1 μg RNA was reverse-transcribed to cDNA with the iScript cDNA Synthesis Kit (Bio-Rad). An ABI 7500 Real-Time PCR instrument (Applied Biosystems) was used to perform the qPCR analysis using iTaq Fast SYBR Green Supermix with ROX (Bio-Rad) in 96-well plates at a final reaction volume of 20 μL. qPCR analysis was carried out in triplicate with the following primer sets: *HSD3B1*, forward, 5′-GTCAAATAGCGTATTCACCTTCTCTTAT-3′; reverse, 5′-GAGGGTGGAGCTTGATGACATCT-3′; *PSA*, forward, 5′-GCATGGGATGGGGATGAAGTAAG-3′; reverse, 5′-CATCAAATCTGAGGGTTGTCTGGA-3′; *TMPRSS2*, forward, 5′-TGGTCCTGGATGATAAAAAAAGTTT-3′; reverse, 5′-GACATACGCCCCACAACAGA-3′; *FKBP5*, forward, 5′-CCCCCTGGTGAACCATAATACA-3′; reverse, 5′-AAAAGGCCACCTAGCTTTTTGC-3′; and *RPLP0* (large ribosomal protein P0, a housekeeping gene), forward, 5′-CGAGGGCACCTGGAAAAC-3′; reverse, 5′-CACATTCCCCCGGATATGA-3′.

For steroid-treated cells, each mRNA transcript was quantitated by normalizing to the housekeeping gene *RPLP0* and to vehicle-treated (fresh media) cells. All gene expression studies were repeated in at least 3 independent experiments.

For Western blot analysis of proteins, cells were lysed in RIPA buffer with protease and phosphatase inhibitors. Primary antibodies used were anti-3βHSD1 antibody (Abcam, 55268; 1:1,000), anti-OGT antibody (Proteintech, 11576; 1:4,000), anti-OGA antibody (Proteintech, 14711; 1:4,000), anti-Elk1 antibody (Proteintech, 27420; 1:4,000), and anti-O-GlcNac antibody (Invitrogen, MA1-072; 1:2,000). Actin was used as a loading control (anti–β-actin antibody, Sigma-Aldrich; 1:5,000). Bands were detected with a chemiluminescence detection system (Thermo Fisher Scientific).

### DHEA metabolism.

For HPLC analysis, cancer cells (~10^5^ cells per well) were seeded and maintained in 24-well plates. After incubation for 48 hours with CAF-CM or RPMI with 10% charcoal-stripped FBS, cells were treated with [^3^H]-DHEA (1,000,000 counts per minute per well; PerkinElmer) and unlabeled DHEA (100 nM final concentration). HPLC was performed as we have previously described (8). In brief, steroid metabolites were separated on a Luna 150 × 4.6 mm, 3 μM C18 reverse-phase column (Phenomenex) using methanol/water gradients at 50°C. The column effluent was analyzed with a β-RAM model 3 in-line radioactivity detector (IN/US Systems Inc.) using Liquiscint scintillation cocktail (National Diagnostics).

For LC-MC/MS, approximately 10^7^ cells were added to 10 cm plates and allowed to settle overnight. Cells were then treated with CAF-CM or RPMI with 10% charcoal-stripped FBS along with DHEA (100 nM final concentration) for 48 hours. Media and lysate were separated by liquid-liquid extraction with methyl tert-butyl ether (Sigma-Aldrich) and separated by reversed-phase chromatography (48).

### Unbiased metabolomics.

RPMI-1640 medium was collected after incubation with CAFs or LNCaP cells for 24 hours. Untargeted metabolomics was performed by injecting 5 μL of each sample onto a 10 cm C18 column (Thermo Fisher Scientific) coupled to a Vanquish UHPLC (Thermo Fisher Scientific) running at 0.2 mL/min using water and 0.1% formic acid as solvent A and acetonitrile and 0.1% formic acid as solvent B. A 30-minute gradient was used. An Orbitrap Q Exactive HF was operated in positive and negative electrospray ionization modes in different LC-MS runs over a mass range of 56 to 850 Da using full MS at a resolution of 120,000. Data-dependent acquisitions were obtained on the pooled quality control sample. The data-dependent acquisition included MS full scans at a resolution of 120,000 and HCD MS/MS scans taken on the 10 most abundant ions at a resolution of 30,000, with dynamic exclusion of 40 seconds and the apex trigger set at 2.0 to 4.0 seconds. The MS2 scans were acquired using stepped NCE energies of 20%, 30%, and 45%. XCMS was used to deconvolute the data using 2.5 ppm consecutive scan error, 6–45 seconds as minimum and maximum peak width, S/N threshold of 10, and span of 0.2 in positive and negative modes for retention time correction. The resulting peak table was further analyzed via MetaboLyzer using 0.7 for ion presence threshold, *P* value threshold of 0.05 using the nonparametric Mann-Whitney *U* test, and false discovery rate correction set at 0.1. Additional details are available in the Supplemental Methods.

### Reporter gene assay.

LNCaP and C42 cells were seeded in 12-well plates (4 × 10^5^ cells per well). Reporter plasmids (containing the 2,000 bp HSD3B1 promoter) were transfected into cells. Renilla plasmid was used in each well as an internal control. Cells were transfected for 24 hours and then treated with CAF-CM for an additional 24 hours. Then, the cells were lysed, and the luciferase activity was analyzed using the Dual-Luciferase Reporter Assay System (Promega) following the manufacturer’s protocol.

### Cell viability assay.

LNCaP and C42 cells (10^4^ cells) were seeded in triplicate in 96-well plates coated with poly-DL-ornithine and allowed to settle overnight. Then, cells were treated with CAF-CM for 72 hours, and cell viability was assayed using WST-1 (Roche) following the manufacturer’s protocol. Viability was normalized to control.

### Immunoprecipitation.

LNCaP and C4-2 cells were seeded in 10 cm dishes and treated with CAF-CM for 48 hours. Immunoprecipitation was performed with the Magnetic IP Kit (Thermo Fisher Scientific, 90409) following the manufacturer’s protocol. Anti–O-GlcNac antibody (Invitrogen, 1:250) was used for immunoprecipitation, and IgG was used as a control. The samples were separated by SDS-PAGE and transferred onto PVDF membranes (Bio-Rad). Western blot experiments were performed with the indicated antibodies and visualized with Super-Signal West Pico Chemiluminescent substrate (Pierce Chemical).

### ChIP analysis.

C4-2 cells were harvested after glucosamine or vehicle treatment for 24 hours. ChIP analysis was performed using the MAGnify Chromatin Immunoprecipitation System (Thermo Fisher Scientific, 492024), following the manufacturer’s instructions. The samples were sonicated 3 times for 10 seconds for 30 cycles (Bioruptor 300) to shear DNA to an average fragment size of 200–400 bp. 5 μg of Elk1 antibodies (Santa Cruz, sc-365876), 2 μg O-GlcNAc antibody (MA1-072), acetyl-histone H3 Lys9 (CST, 9649S), and acetyl-histone H3Lys27 (CST, 8173) were used for the assay.

Each sample was analyzed in triplicate by real-time PCR using specific primers, as described in the Supplemental Methods. Enrichment was determined by using the 2−ΔCT method, comparing with vehicle. Primer specificity was confirmed by evaluation of dissociation curves and independently analyzing amplified product on an agarose gel.

### Mouse xenograft studies.

All NOD/SCID/γ male mice (6–8 weeks old) were purchased from The Jackson Laboratory. Mice were subcutaneously injected with 10^7^ sgControl or Elk1-KO (sgElk1) C4-2 cells with or without 10^7^ CAFs (total injection volume 100 μL). Once tumors reached 100 mm^3^ (length × width × height × 0.52), mice were surgically orchiectomized and implanted with 5 mg 90-day sustained-release DHEA pellets (Innovative Research of America) to mimic human adrenal DHEA production in patients with CRPC. The number of mice in each group was as follows: sgControl (*n* = 6), sgElk1 (*n* = 5), sgControl/CAF (*n* = 5), and sgElk1/CAF (*n* = 9).

At study completion, mouse serum and xenografts were collected and flash-frozen for steroid analysis, as described previously with slight modifications (21, 49). At least 24 mg tumor tissue was homogenized with 1 mL LC-MS grade water (Thermo Fisher Scientific) using a homogenizer. The mixture was then centrifuged (15,000*g*), and 800 μL supernatant was transferred to a glass tube. Internal standards (25 μL of d_2_-DHEA, ^13^C_3_-AD, and d_3_-T) were then added. Steroids and the internal standard were extracted with 4 mL methyl tert-butyl ether (Sigma Aldrich), evaporated under a stream of nitrogen gas, and reconstituted in 200 μL methanol/water (50:50) prior to LC-MS analysis. For serum, 20 μL was subjected to direct protein precipitation with 180 μL methanol containing the internal standard (d_2_-DHEA, ^13^C_3_-AD, and d_3_-T). The mixture was then centrifuged (15,000 × g) for 10 minutes at 4°C, and the supernatant was transferred to HPLC vials for LC-MS analysis.

### Clinical analyses.

Tyramide signal amplification multiplexed fluorescence was used to stain radical prostatectomy prostate cancer tissues. Sections were incubated with anti-3βHSD1 (Abcam, 55268; 1:200), anti-Elk1 (Thermo Fisher Scientific, MA5-15310; 1:100), anti-SMA (Abcam, 158031; 1:200) antibodies, and DAPI. The results were photographed under an Olympus IX71 fluorescence microscope. Visiopharm software was used to quantify the stained areas and stain density. The Memorial Sloan Kettering Cancer Center (MSKCC) prostate cancer database was queried using cBioPortal and analyzed (GEO GSE21032). Data from a metastatic CRPC data set (GSE77930) were analyzed after interbatch correction.

### Statistics.

Statistical analyses were performed with GraphPad Prism software. In general, for mouse xenograft studies, progression-free survival was analyzed by Kaplan-Meier analysis, and curves were compared among groups with a log-rank test. The correlation between 2 genes was analyzed using the Pearson correlation test. For other comparative analyses, unless otherwise noted, a 2-tailed *t* test or 1-way ANOVA test was used, and data are shown as mean ± SEM. *P* < 0.05 was considered statistically significant.

### Study approval.

All mouse studies were performed under a protocol approved by the Institutional Animal Care and Use Committee of the Cleveland Clinic Lerner Research Institute. All human tissues were obtained under Shanghai General Hospital review board–approved protocols. Fresh prostatectomy tumor tissue was obtained from patients with prostate cancer at the Department of Urology, Shanghai General Hospital.

## Author contributions

NS and DC conceived the study. DC, JL, and ZZ planned and performed experiments, analyzed data, and wrote the manuscript. MB, MA, RT, and YMC performed experiments. MG, BW, AH, JM, and SV provided help with experiments. BH provided clinical samples utilized in this study. NS supervised the study. All authors read and approved the manuscript.

## Supplementary Material

Supplemental data

## Figures and Tables

**Table 1:**
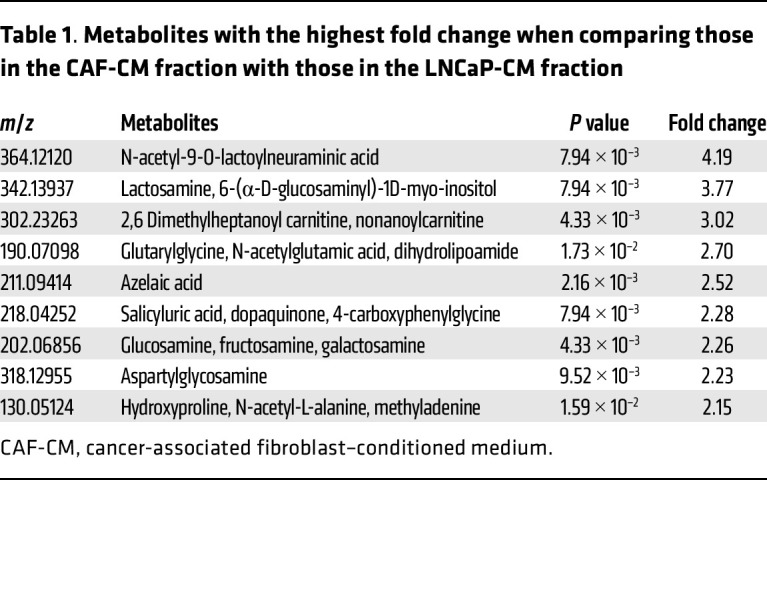
Metabolites with the highest fold change when comparing those in the CAF-CM fraction with those in the LNCaP-CM fraction

## Version 1 04/03/2023

Electronic publication
