# Supplementary material for: Cancer-associated fibroblast-secreted glucosamine alters the androgen biosynthesis program in prostate cancer via HSD3B1 upregulation
Source: J Clin Invest. 2023 Apr 3;133(7):e161913. doi: 10.1172/JCI161913 (PMC10065083; doi:10.1172/JCI161913)
Supplement: Supplemental data [file jci-133-161913-s143.pdf]

Supplemental figures

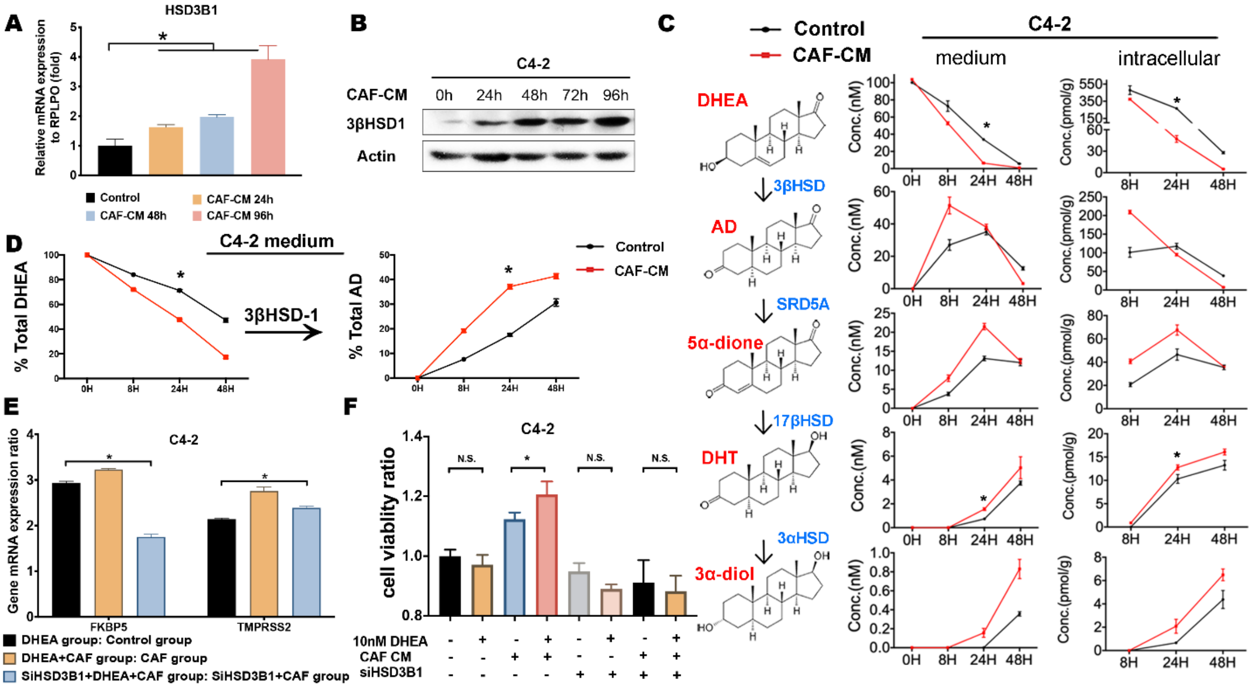

Figure S1. CAFs increase the conversion from DHEA to active androgens in C4-2 cells by increasing 3βHSD1 expression and enzyme activity. (**A**) mRNA and protein (**B**) expression of *HSD3B1* and 3βHSD1 in C4-2 cells treated with CAF-conditioned medium for the indicated times. Gene and protein expression was normalized to RPLP0 and β-actin, respectively. (**C**) C4-2 cells were treated with CAF-CM for 48 hours followed by 100 nM DHEA for the indicated times. Downstream androgens in intracellular and media samples were quantitated by mass spectrometry. (**D**) C4-2 cells were treated with CAF-CM for 48 hours followed by [^{3}H]-DHEA for the indicated times followed by extraction of steroids from medium and quantitated by HPLC. (**E**) Gene expression of AR target genes FKBP5 and TMPRSS2 expression in C4-2 cells expressing control or HSD3B1 siRNA and treated with 10 nM DHEA and CAF-CM for 48 hours. Expression was normalized to untreated cells (not shown) and RPLP0 was used as a loading control. (**F**) Cell viability of C4-2 control or HSD3B1 siRNA cells treated with 10 nM DHEA along with control

media or CAF-CM. Viability was normalized to the untreated control. Unless otherwise noted, error bars represent the SEM, and significance was calculated using two-tailed t-tests or one-way ANOVA. * $P < 0.05$, N.S. not significant.

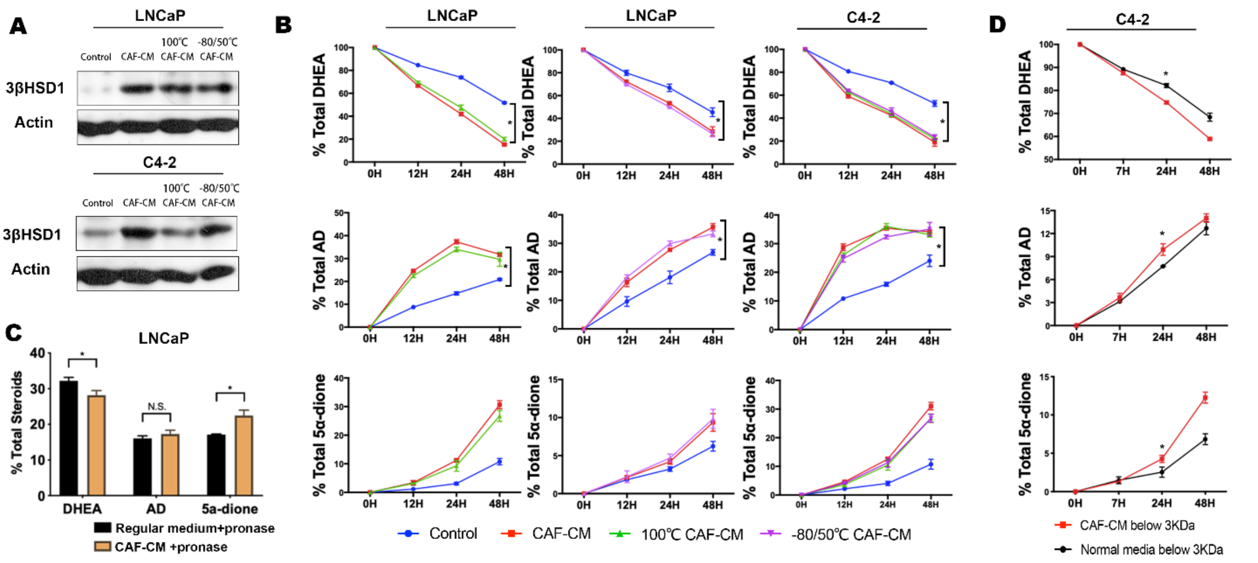

Figure S2. Effect of CAF-conditioned medium is not reversed when treated with heat inactivation or proteinases. (**A**) Protein expression of 3βHSD1 in LNCaP (top panel) and C4-2 (bottom panel) cells treated for 48 hours with CAF-CM, boiled conditioned media (100°C CAF-CM), or freeze-thawed media (-80°C/50°C CAF-CM). β-actin was used as a loading control. (**B**) HPLC analysis of steroids in media of LNCaP and C4-2 cells treated with CAF-CM, boiled conditioned media, or freeze-thawed media for 48 hours followed by 100 nM [^{3}H]-DHEA for the indicated times. (**C**) HPLC analysis of steroids in media of LNCaP cells treated with CAF-CM or conditioned media pre-incubated for 24 hours with Pronase, a protease that degrades denatured and native proteins. (**D**) HPLC analysis of steroids in media of LNCaP cells treated with CAF-CM or fractionated conditioned media centrifuged using filters with a 3-kDa molecular weight cut-off. Error bars represent the SEM, and significance was calculated using two-tailed t tests or one-way ANOVA (**C**). * $P < 0.05$, N.S. not significant.

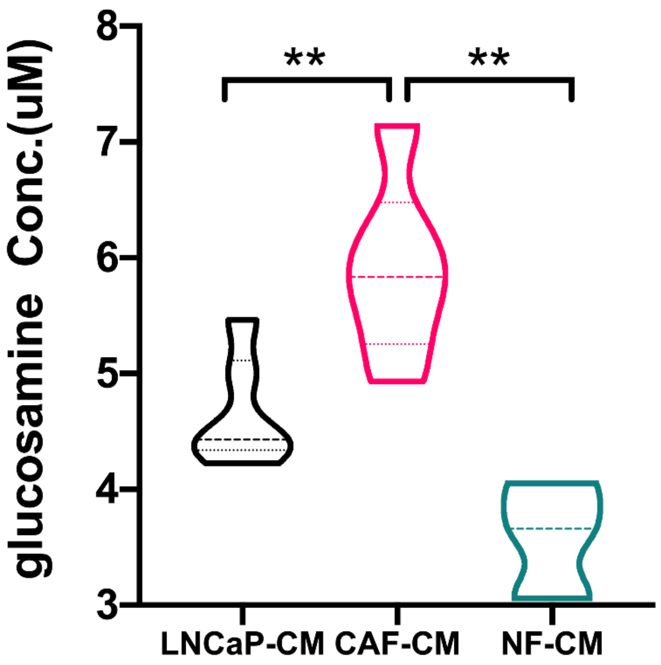

Figure S3. Glucosamine in conditioned media (CM). LNCaP, cancer-associated fibroblasts (CAF) and normal prostate fibroblasts (NF) were cultured in 10 cm dishes for 48 hours and glucosamine concentrations were assessed in triplicate using mass spectrometry. Significance was calculated using two-tailed t- tests. ** $P < 0.01$

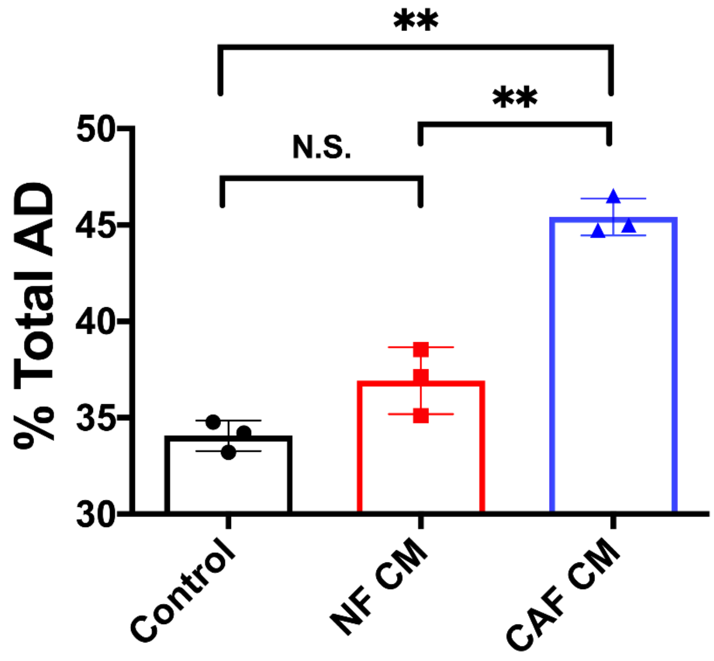

Figure S4. The effect of normal fibroblast (NF) and cancer-associated fibroblast (CAF) conditioned media on AD synthesis. LNCaP cells were cultured in RPMI-1640 medium (control), or NF-CM or CAF-CM for 48 hours. [^{3}H]-AD synthesis from DHEA was tested by HPLC as described previously. Significance was calculated using one-way ANOVA and error bars represent SEM. ** $P < 0.01$

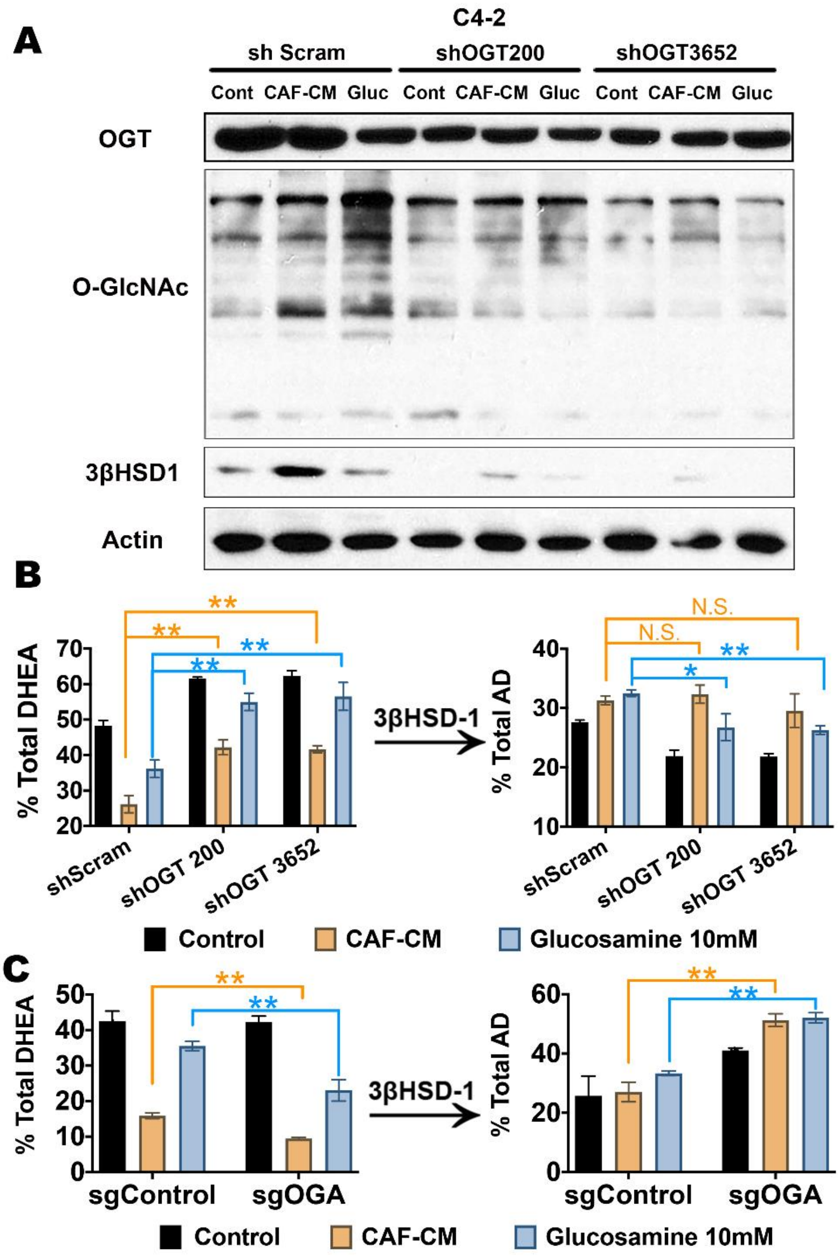

Figure S5. High O-GlcNAcylation by CAF-CM and glucosamine induces 3βHSD1 expression and activity in C4-2 cells. (**A**) Western blot analysis of O-GlcNAcylation, OGT, and 3βHSD1 levels in C4-2 cells expressing two shRNAs targeting OGT (OCG200 and OGT 3652) or scrambled shRNA and treated with CAF-CM or 10 mM glucosamine for 48 hours. (**B**) HPLC analysis of DHEA metabolism in C4-2 cells expressing scrambled shRNA, shOCG200, or shOGT 3652 treated with CAF-CM or 10 mM glucosamine for 48 hours followed by the addition of [^{3}H]-DHEA (100 nM) for the indicated times. Significance was calculated using two-

tailed t- tests or one-way ANOVA and error bars represent SEM. * $P < 0.05$, ** $P < 0.01$, N.S. = not significant.

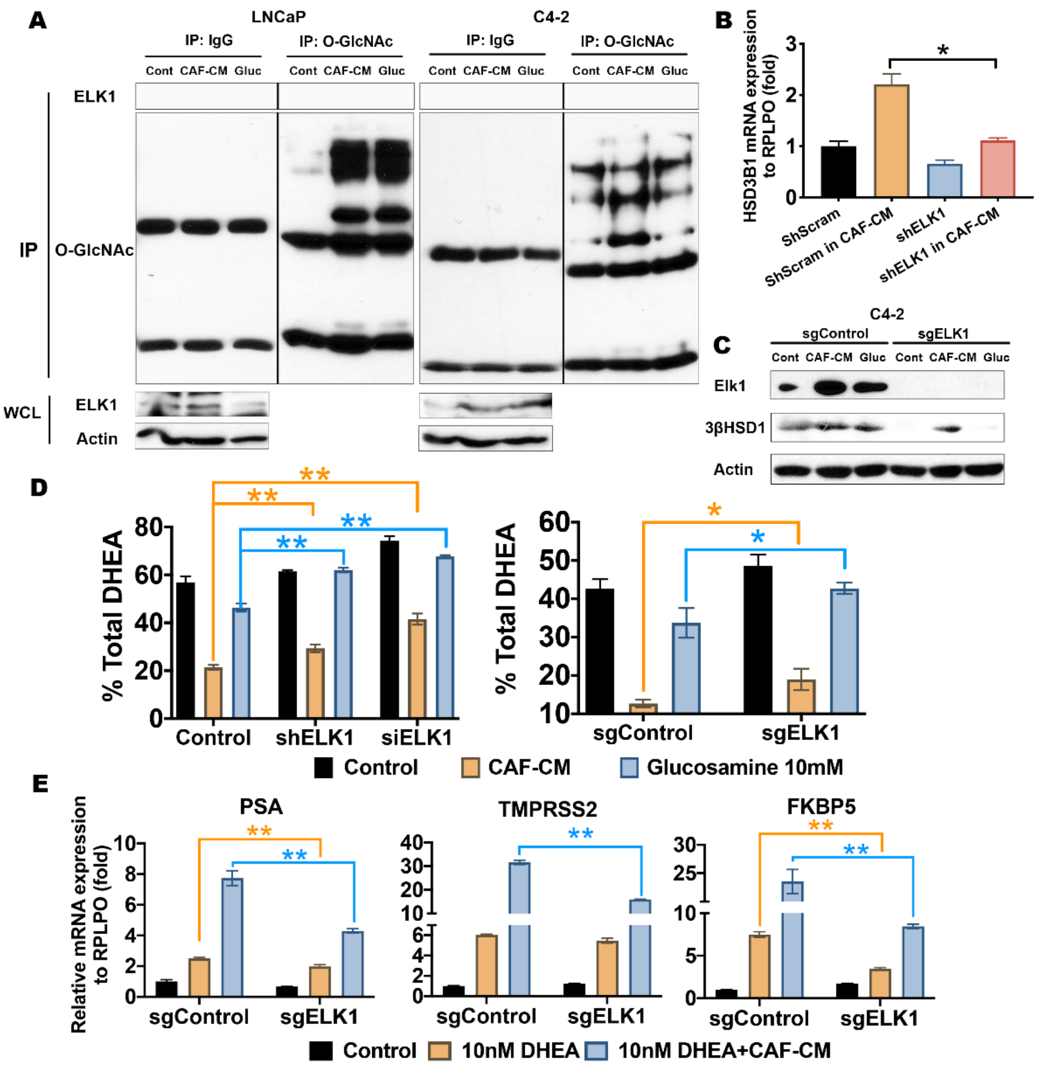

Figure S6. High O-GlcNAcylation by CAF-CM or glucosamine induces 3βHSD1 expression and enzyme activity in C4-2 cells. (**A**) LNCaP and C4-2 cells were treated with CAF-CM or 10 mM glucosamine for 48 hours. Co-immunoprecipitation was performed using a non-targeting IgG control or O-GlcNAcylation antibody. Western blotting was performed using antibodies against ELK1, O-GlcNAcylation, and β-actin on whole cell lysate (WCL) and IP samples. (**B**) Gene expression of HSD3B1 in C4-2 cells expressing shRNA targeting *ELK1* (shELK1) or scrambled control (shScramb) and treated with CAF-CM for 48 hours. (**C**) Protein expression of 3βHSD1in C4-2 cells transduced with control (sgControl) or sgRNA targeting *ELK1* (sg*ELK1*) after 48-hour treatment with CAF-CM or 10 mM glucosamine. (**D**) HPLC analysis of steroids in media of siRNA

and shRNA knockdown (left panel) or transduced ELK1 sgControl and *ELK1* KO (right panel) C4-2 cells. Cells were treated with CAF-CM or glucosamine for 48 hours followed by [^{3}H]-DHEA (100 nM) for 48 hours. (**E**) Gene expression of PSA, TMPRSS2, and FKBP5 in sgControl and *ELK1* KO C4-2 cells treated with 10 nM DHEA along with CAF-CM or glucosamine for 48 hours. Significance was calculated using two-tailed t- tests or one-way ANOVA and error bars represent SEM.* $P < 0.05$, ** $P < 0.01$.

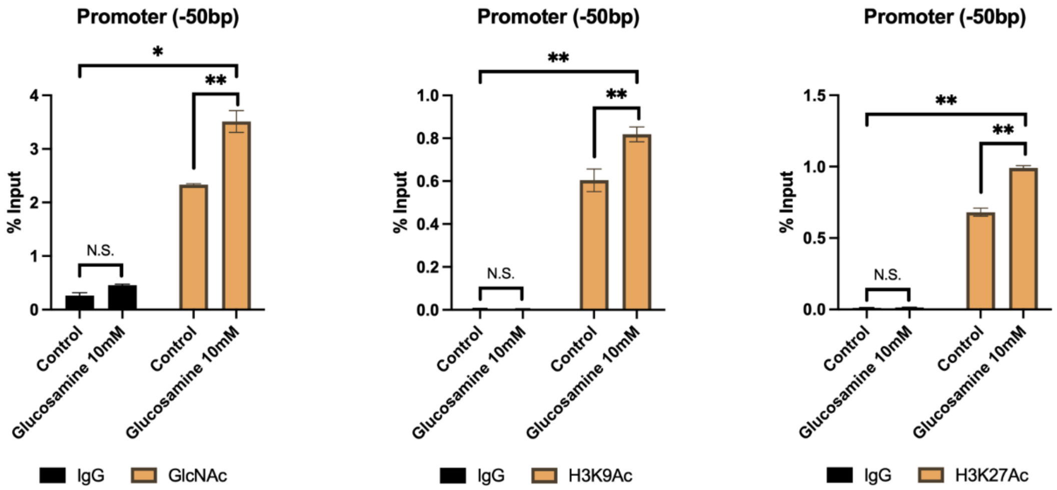

Figure S7. Glucosamine increases chromatin GlcNAc and acetylation on the Elk1 binding site of HSD3B1 promoter. ChIP assay of O-GlcNAc, H3K9Ac and H3K27Ac. C4-2 cells were treated with 10 mM glucosamine for 24 hours prior to the ChIP assays.

Supplemental Methods

Metabolomics procedure and analysis

Processing Methods

Fractions of 12 cell media samples (6 CAF-conditioned medium and 6 LNCaP-conditioned medium) with desired activity were prepared for untargeted metabolomics by diluting each sample 1:1 in chilled methanol containing 5 internal standards as listed in **Table 1**. The samples were then centrifuged at 14,000*g* for 20 minutes to precipitate out the protein pellet. The supernatant was recovered and subjected to LC-MS analysis. One-microliter aliquots taken from each sample were pooled and this QC standard was analyzed every 5 injections.

Table 1. Internal standards

**Name**	**Adduc**	**Observed**	**Final**
Betaine-d9	H+	127.143	21.15
Carnitine-d9	H+	171.169	20.65
Ornithine-d6	H+	139.135	5.25
Valine- 13C3	H+	123.092	20.45
Tyrosine- 13C, 15N	H+	192.108	5.25
Estrone- 13C3	H+	274.341	30
Cholesterol- 13C3	H+	390.635	30

The untargeted metabolomics was performed by injecting 5 µL of each sample onto a 10-cm C18 column (Thermo Fisher CA) coupled to a Vanquish UHPLC running at 0.2mL/min using water and 0.1% formic acid as solvent A and acetonitrile and 0.1% formic acid as solvent B. The 30-min gradient used is shown in **Table 2.** The Orbitrap Q Exactive HF was operated in positive

and negative electrospray ionization modes in different LC-MS runs over a mass range of 56-850 Da using full MS at 120,000 resolution. Data-dependent acquisitions were obtained on the pooled QC sample. This included MS full scans at a resolution of 120,000 and HCD MS/MS scans taken on the top 10 most abundant ions at a resolution of 30,000 with dynamic exclusion of 40 seconds and the apex trigger set at 2.0 to 4.0 seconds. The resolution of the MS2 scans were taken at a stepped normalized collision energy of 20.0, 30.0 and 45.0.

Table 2. HPLC gradient used

Time (min)	% B	Flow (mL/min)
0.2	5	0.2
5	10	0.2
20	65	0.2
26	95	0.2
28	95	0.2
28.1	5	0.2
30	5	0.2

Data Analysis

XCMS was used to deconvolute the data using 2.5 ppm consecutive scan error, 6 to 45 seconds as minimum and maximum peak width, S/N threshold of 10, and span of 0.2 in positive and negative

modes for retention time correction. The resulting peak table was further analyzed via MetaboLyzer. Briefly, the ion presence threshold was set at 0.7 in each study group. Data were then log-transformed and analyzed for statistical significance via parametric Mann-Whitney U-test; significance was set at *P* <0.05). Ions present in just a subset of samples were analyzed as categorical variables for presence status via Fisher's exact test. All *P*-values were corrected via the Benjamini-Hochberg step-up procedure for false discovery rate (FDR) correction, which was set at 0.2.

The data were then utilized for principal components analysis, putative identification assignment, and pathway enrichment analysis via KEGG. In this dataset, 16,523 spectral features were detected in both electrospray ionization modes, from which 2,144 features were putatively assigned an identification in HMDB within a pre-defined 7ppm m/z error window. Also, the MS/MS spectra of 194 of these features matched with a score of greater than 50% to unique compounds on the mzCloud database (**Table 3**).

Table 3. Examples of ions with statistically significant changes in CAF samples compared to LNCaP samples

m/z	RT (min)	Putative ID	FDR-adjusted *P* values	Fold-change*	ESI mode

385.34634	26.21	7-α-hydroxcholesterol	0.15	0.78	POS
399.32574	27.01	7-α-24S-dihydroxy-4-cholesten-3-one	0.15	0.78	POS
311.29435	24.64	cis-13-eicosenoic acid	0.15	1.18	POS
285.22128	25.54	17-α-methyltestosterone	0.17	1.19	POS
175.02574	1.06	glucuronic acid	0.18	2.03	NEG
146.04637	1.06	glutamic acid	0.18	0.44	NEG
118.05104	1.02	homoserine	0.18	0.80	NEG

*Fold change was calculated by taking the ratio of average relative ion abundance in the CAF samples to that in the LN samples.

ChIP primers

The Elk-1 binding motifs were predicted by JASPAR. Primers on the upstream of HSD3B1 translation start site were designed as below.

-50-f	CTGCAGCATTAGGATGGGACT
-50-r	CTGGCCCAACCCTTATCACT
-800-f	GTCATTGGGGCATACAACCAC
-800-r	AGAGCCCTGGGTTCCTAGATG
-2000-f	TTGTAGCACAAGGCCAACCT
-2000-r	CTTGCCCTCCTCCATGTTGT
